# Body Silhouette Trajectories Over the Lifespan and Insomnia Symptoms: The Paris Prospective Study 3

**DOI:** 10.1038/s41598-018-38145-7

**Published:** 2019-02-07

**Authors:** Q. Lisan, M. Tafflet, F. Thomas, P. Boutouyrie, C. Guibout, J. Haba-Rubio, R. Climie, M. C. Périer, T. Van Sloten, B. Pannier, P. Marques-Vidal, X. Jouven, J. P. Empana

**Affiliations:** 1 Université Paris Descartes, Sorbonne Paris Cité, Faculté de Médecine, Paris, France; 2 INSERM, UMR-S970, Paris Cardiovascular Research Center, Department of Epidemiology, Paris, France; 3 AP-HP, Georges Pompidou European Hospital, Department of Head and Neck surgery, Paris, France; 4 Preventive and Clinical Investigation Center, Paris, France; 5 AP-HP, Georges Pompidou European Hospital, Department of Pharmacology, Paris, France; 6 Center for Investigation and Research in Sleep, Lausanne University Hospital, Lausanne University, Lausanne, Switzerland; 7 Menzies Institute for Medical Research, University of Tasmania, Hobart, Australia; 8 CARIM School for Cardiovascular Diseases, Maastricht University, Maastricht, The Netherlands; 9 Department of Internal Medicine, Maastricht University Medical Centre, Maastricht, The Netherlands; 10 Department of medicine, Service of internal medicine, Lausanne University Hospital, Lausanne, Switzerland; 11 AP-HP, Georges Pompidou European Hospital, Cardiology Department, Paris, France

## Abstract

Insomnia symptoms are highly prevalent and associated with several adverse medical conditions, but only few determinants, including non-modifiable ones, have been highlighted. We investigated associations between body silhouette trajectories over the lifespan and insomnia symptoms in adulthood. From a community-based study, 7 496 men and women aged 50–75 years recalled their body silhouette at age 8, 15, 25, 35 and 45, and rated the frequency of insomnia symptoms on a standardized sleep questionnaire. An Epworth Sleepiness Scale ≥11 defined excessive daytime sleepiness (EDS). Using a group-based trajectory modeling, we identified five body silhouette trajectories: a ‘lean-stable’ (32.7%), a ‘heavy-stable’ (8.1%), a ‘moderate-stable’ (32.5%), a ‘lean-increase’ (11%) and a ‘lean-marked increase’ (15.7%) trajectory. In multivariate logistic regression, compared to the ‘lean-stable’ trajectory, the ‘lean-marked increase’ and ‘heavy-stable’ trajectories were associated with a significant increased odd of having ≥1 insomnia symptoms as compared to none and of having a proxy for insomnia disorder (≥1 insomnia symptom and EDS). The association with the ‘lean-marked increase' trajectory’ was independent from body mass index measured at study recruitment. In conclusion, increasing body silhouette over the lifespan is associated with insomnia symptoms in adulthood, emphasizing the importance of weight gain prevention during the entire lifespan.

## Introduction

Insomnia symptoms such as difficulty in initiating or maintaining sleep, or early morning awakening, are the most common sleep complaints^1,2^ and are reported in up to 50% in adults^3–5^. The prevalence of insomnia symptoms is expected to rise, due to societal factors (24 h/7 days modern societies) and to changes in occupational factors such as increased work stress and shiftwork, and reduced time spent asleep^6–8^. Insomnia symptoms have been associated with an increased risk of hypertension, heart failure, coronary heart disease and mortality^9–11^. Moreover, insomnia symptoms are associated with multiple impairments in daily living – commonly referred to as insomnia disorder^3,12^ – such as home and work accident^13^, absenteeism and decreased work productivity, and impaired quality of life^2,14,15^.

Given these important social and medical consequences, prevention of insomnia symptoms represents a public health challenge. This is even more important as the affordability and efficacy of proposed treatments, including cognitive behavioral therapy and pharmacologic treatments, have not been firmly established^16,17^. A better understanding of the risk factors for insomnia symptoms is a pre requisite to develop preventive strategies of insomnia symptoms. So far, few determinants of insomnia symptoms have been reported, including among others aging, female gender, lower socioeconomic status, shift work or depression^3,5,18–22^.

While increased adiposity – an epidemic and modifiable risk factor – has been associated with sleep disorders such as excessive daytime sleepiness^23,24^ or obstructive sleep apnea^25^, inconsistent and contradictory associations have been reported with insomnia symptoms^21,26,27^. This discrepancy may partly rely on the cross-sectional design of most studies^21,26,27^, or the limited number of repeated measures of adiposity over time among longitudinal studies^28,29^. Indeed, adiposity changes over time, especially at critical periods such as during puberty and menopause. Hence, repeated measures of adiposity over a long time period within the same individual may provide more accurate information regarding its evolution and its potential association with insomnia symptoms. In large epidemiological studies, repeated assessments of objective measures of adiposity are rarely available, in particular regarding childhood and adolescent periods. Under these circumstances, self-reported body silhouettes at different ages may represent a simple and reliable alternative^30,31^. By combining recall of body shapes at different ages within the same subject, it is possible to construct body silhouette trajectories. This is of public health importance since body silhouette trajectories have been recently associated with incident type 2 diabetes, cancer and mortality^32–35^.

Therefore, in this study, we sought to investigate the potential associations between body silhouette trajectories over the lifespan from childhood to adulthood and insomnia symptoms in adulthood.

## Results

### Characteristics of the study population

The study population comprised 61.5% of men and the median age was 59.2 years (interquartile range 54.5–63.6 years). Overall, 61.4% reported at least one insomnia symptom and 11.7% reported insomnia disorder (proxy). Furthermore, 55.1% of the participants had DMS, 26.5% EMA and 18% DIS.

**Figure 1:**
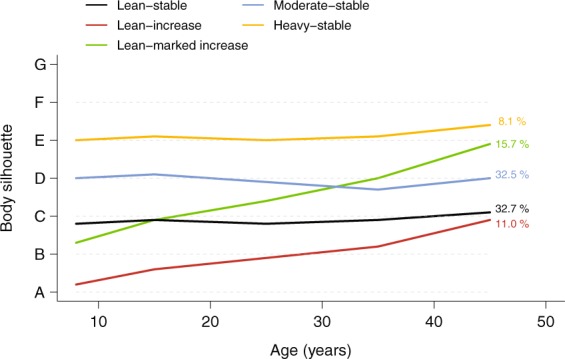
Trajectories of body silhouette. Note: percentages represent the proportion of the population in each trajectory.

Among the five body silhouette trajectories (Fig. 1), 8.1% of the participants had a ‘heavy-stable’ trajectory, 32.5% a ‘moderate-stable’ and 32.7% a ‘lean-stable’; 11% had a ‘lean-increase’ trajectory and 15.7% a ‘lean-marked increase’ trajectory.

The main characteristics of the study population according to the body silhouettes trajectories are reported in Table 1. In particular, the presence of at least one insomnia symptom and the presence of insomnia disorder (proxy) were more frequently observed among participants belonging to the ‘lean-marked increase’ and ‘heavy-stable’ trajectories.

**Table 1:** Study participants’ characteristics according to the body silhouette trajectory.

	Body silhouette trajectory				
	Lean-stable N = 2 438	Lean-increase N = 823	Lean-marked increase N = 1 176	Moderate-stable N = 2 449	Heavy-stable N = 610
***Insomnia symptoms and proxy for insomnia disorder***					
At least one insomnia symptom	1 445 (59.3)	512 (62.2)	750 (63.8)	1 494 (61.0)	405 (66.4)
Insomnia disorder (proxy)	241 (9.9)	92 (11.2)	175 (14.9)	271 (11.1)	98 (16.1)
***Socio-demographics data***					
Male gender	1 515 (62.1)	588 (71.4)	879 (74.7)	1 343 (54.8)	285 (46.7)
Age (years)	59.7 ± 6.07	60.5 ± 6.32	59.3 ± 6.27	59.5 ± 6.30	59.0 ± 6.07
Body mass index (kg/m^2^)	23.7 ± 2.95	24.0 ± 3.11	26.8 ± 3.47	25.2 ± 3.46	27.8 ± 4.36
Birth weight categories*					
<2.5 kg	111 (5.3)	61 (9.4)	68 (7.1)	83 (4.0)	20 (3.9)
2.5–4 kg	1 805 (86.9)	542 (83.8)	803 (84.1)	1 775 (85.4)	411 (80.4)
>4 kg	161 (7.8)	44 (6.8)	84 (8.8)	220 (10.6)	80 (15.7)
Education level					
No graduation	63 (2.61)	33 (4.06)	70 (5.99)	70 (2.88)	19 (3.14)
Under high school diploma	498 (20.7)	189 (23.2)	310 (26.5)	528 (21.7)	167 (27.6)
≥high school diploma	1 849 (76.7)	591 (72.7)	789 (67.5)	1 833 (75.4)	420 (69.3)
Current smoker	312 (12.8)	106 (12.9)	145 (12.4)	358 (14.6)	102 (16.8)
Coffee consumption					
Never	426 (17.6)	160 (19.6)	165 (14.2)	364 (15.0)	81 (13.4)
1–4 cups a day	1 842 (76.0)	598 (73.1)	898 (77.1)	1 865 (76.7)	458 (75.6)
≥5 cups a day	157 (6.47)	60 (7.33)	101 (8.68)	204 (8.38)	67 (11.1)
Living alone	588 (24.3)	189 (23.0)	251 (21.4)	616 (25.3)	173 (28.5)
Alcohol consumption					
Never	237 (9.90)	79 (9.77)	122 (10.5)	256 (10.6)	68 (11.3)
1–2 drinks per day	1 855 (77.5)	610 (75.4)	849 (73.1)	1 871 (77.5)	484 (80.1)
≥3 drinks per day	302 (12.6)	120 (14.8)	191 (16.4)	287 (11.9)	52 (8.61)
Score of physical activity	6.26 ± 1.41	6.17 ± 1.43	6.19 ± 1.41	6.24 ± 1.42	6.26 ± 1.53
***Comorbid conditions***					
Depression	168 (6.94)	61 (7.43)	105 (8.94)	207 (8.48)	77 (12.7)
Stress (PSS4 score)	3.62 ± 2.70	3.70 ± 2.76	3.82 ± 2.69	3.75 ± 2.71	4.16 ± 2.93
Prevalent CVD	33 (1.36)	20 (2.44)	32 (2.73)	49 (2.01)	10 (1.64)
Hypertension	694 (28.6)	296 (36.3)	469 (40.0)	839 (34.5)	227 (37.4)
Diabetes	46 (1.89)	33 (4.01)	77 (6.58)	77 (3.16)	21 (3.47)
***Sleep related variables***					
Excessive daytime sleepiness	363 (14.9)	129 (15.7)	230 (19.6)	382 (15.7)	120 (19.7)
Night working	37 (1.52)	17 (2.07)	21 (1.79)	30 (1.23)	8 (1.31)
SDB (proxy)	26 (1.07)	12 (1.47)	105 (8.98)	97 (3.99)	89 (14.8)
Use of sleep related medications	77 (4.14)	30 (4.72)	52 (5.45)	87 (4.51)	25 (4.80)
Sleep duration (hours)					
6–9 h per night	2 047 (87.0)	664 (84.9)	948 (83.3)	2 037 (86.4)	494 (85.5)
<6 h per night	277 (11.8)	106 (13.6)	173 (15.2)	290 (12.3)	80 (13.8)
>9 h per night	29 (1.23)	12 (1.53)	17 (1.49)	31 (1.31)	4 (0.69)

Abbreviations: CVD: cardiovascular disease, SDB: sleep disordered breathing.

Note: values are number of participants (percentages) or mean ± standard deviation.

*Available in n = 6268 subjects

### Associations of body silhouette trajectories with insomnia symptoms and insomnia disorder

The unadjusted and multivariable adjusted ORs of body silhouettes trajectories for the presence of at least one insomnia symptom and for the presence of insomnia disorder (proxy) are presented in Table 2 (OR of the covariates are shown in Table S1). In multivariate analysis and compared to the ‘lean-stable’ trajectory, the ‘lean-marked increase’ trajectory was associated with the presence of at least one insomnia symptom (OR = 1.36; 95% CI 1.15–1.61) and with the presence of insomnia disorder (OR = 1.67; 95% CI 1.32–2.12). Similarly, the ‘heavy-stable’ trajectory was associated with both ≥1 insomnia symptom (OR 1.28, 95% CI 1.03–1.58) and the proxy for insomnia disorder (OR 1.50, 95% CI 1.12–2.01).

**Table 2:** Multivariate associations of body silhouette trajectories for insomnia symptoms and for insomnia disorder (proxy).

	Outcome			
	≥1 symptom versus 0		Proxy for insomnia disorder (≥1 symptom and EDS)	
	Unadjusted	Fully adjusted	Unadjusted	Fully adjusted
***Body silhouette trajectory***				
Lean-stable	1 (reference)	1 (reference)	1 (reference)	1 (reference)
Lean-increase	1.13 (0.96–1.33)	1.17 (0.97–1.42)	1.15 (0.89–1.47)	1.10 (0.81–1.48)
Lean-marked increase	1.21 (1.05–1.40)	1.36 (1.15–1.61)	1.60 (1.29–1.96)	1.67 (1.32–2.12)
Moderate-stable	1.08 (0.96–1.21)	1.09 (0.95–1.25)	1.14 (0.95–1.36)	1.08 (0.88–1.34)
Heavy-stable	1.36 (1.13–1.64)	1.28 (1.03–1.58)	1.75 (1.35–2.24)	1.50 (1.12–2.01)

Abbreviations: OR: odd ratio, CI: confidence interval.

Note: fully adjusted models are adjusted for age, sex, education level, coffee and alcohol consumptions, living alone, physical activity, depression, stress and sleep medications.

The results of the sex-stratified analysis are reported in Table S2. In both men and women, the ‘lean-marked increased’ trajectory was significantly associated with the presence of at least one insomnia symptom, and with the presence of insomnia disorder (proxy). The effect size of the association with at least one insomnia symptom was stronger in women compared to men. The ‘heavy stable’ trajectory was significantly associated with the presence of at least one insomnia symptom in women, and with the presence of insomnia disorder (proxy) in men.

The results of the analysis between body silhouette trajectories and each insomnia symptom are presented in Table S3. Statistically significant associations were found for the ‘lean-marked increased’ trajectory with DMS and EMA, and with the ‘lean increased’ trajectory with DIS.

### Supplementary analyses

As presented in the Table S4, when considering body silhouette at age 45, only silhouettes E and G (indicating high level of adiposity) were associated with ≥1 insomnia symptom and with proxy for insomnia disorder.

Associations of the ‘lean-marked increase’ trajectory with ≥1 insomnia symptom (OR 1.33, 95% CI 1.12–1.58) and with the proxy for insomnia disorder (OR 1.55, 95% CI 1.22–1.97) remained unchanged after adjusting for a proxy for SDB.

When considering the score of insomnia symptoms, the ‘lean-increase’, ‘lean-marked increase’ and ‘heavy-stable’ trajectories were all associated with a higher score of insomnia symptoms (Table S5).

Compared to a BMI between 18.5 and 25 kg/m^2^ at study recruitment, a BMI > 30 kg/m^2^ was significantly associated with the proxy for insomnia disorder (OR 1.68, 95% CI 1.29–2.17) but not with the presence of at least one insomnia symptom (OR 1.17, 95% CI 0.96–1.43) in multivariate analysis. Neither overweight (BMI from 25 to 29.9 kg/m^2^) nor underweight (BMI < 18.5 kg/m^2^) was related to any insomnia outcome.

After adjusting the analysis of body silhouette trajectories for BMI at study recruitment, the ‘heavy-stable’ trajectory was significantly associated with ≥1 insomnia symptom and was no longer associated with insomnia disorder (proxy). Associations of the ‘lean-marked increase’ trajectory with ≥1 insomnia symptom and with insomnia disorder (proxy) remained significant. In this analysis, only BMI > 30 kg/m^2^ as compared to BMI from 18.5 to 25 kg/m^2^ was associated with the proxy for insomnia disorder (OR 1.49, 95% CI 1.12–1.95) but not for the presence of at least one insomnia symptom (Table S6).

Birth weight was available in 6 268 participants of the analytic sample. Additional adjustment for birth weight categories did not affect the associations of body silhouette trajectories with insomnia outcomes, and in this model, birth weigh categories < 2.5 kg or above 4 kg as compared to 2.4–4 kg were not related to any insomnia outcomes (not shown).

Similarly, further adjustment for type 2 diabetes did not affect associations of body silhouette trajectories with insomnia outcomes (not shown).

## Discussion

Using a lifetime approach of self-reported body silhouettes from childhood (8 years of age) to adulthood (45 years) in 7 496 community-dwelling men and women, we first identified five body silhouettes trajectories including ‘heavy-stable’, ‘moderate-stable’ and ‘lean-stable’; and a ‘lean increase’ and ‘lean-marked increase’ trajectory group. Second, we found that participants with a ‘lean-marked increase’ trajectory, and to a lesser extent those with a ‘heavy-stable’ trajectory, had a significant increased odds of having insomnia symptoms and insomnia disorder (proxy) in adulthood compared to those with a ‘lean-stable’ trajectory. These associations were independent of major confounding factors.

In cross-sectional studies, in one study the increased odds of insomnia symptoms associated with obesity (BMI ≥ 30 kg/m^2^) disappeared after adjustment for confounders such as depression^21^, while in another, obesity was related to lower odds of insomnia symptoms in women but not in men^27^.

Regarding the few existing prospective studies, in the Penn State Sleep Cohort obesity at baseline showed a marginally significant 47% increased odds of incident chronic insomnia over a 7.5-year follow-up after controlling for sociodemographic and behavioral factors, but this association attenuated and was no longer significant in fully-adjusted analysis including comorbidities^36^. In the Helsinki Health Study, higher BMI in women was associated with persistent (occasional and frequent) insomnia symptoms, and with incident and an increase in insomnia symptoms over two examinations^26^. Similar associations were found in men but in general with weaker effect size. There were however some counterintuitive findings in that study such that obese and severely obese were more likely to report full or partial remission of insomnia symptoms, whereas men with a BMI between 30 and 34.99 kg/m^2^ were less likely to report persistent occasional insomnia symptoms compared to normo weighted men. However, these latter findings were based on small groups and require replication, as acknowledged by the authors^26^.

Studies investigating changes in BMI with the risk of insomnia symptoms are even scarcer. In one study conducted exclusively in men (n = 2 602), change in BMI over 10 years was neither associated with persistent or incident insomnia symptoms nor with remission of insomnia symptoms, contrary to higher baseline BMI^28^. In a smaller study (n = 464) exploring the psychosocial and biological determinants of change in sleep quality assessed three times over 1 year, subjects with incident insomnia symptoms and with incident insomnia syndrome had moderate increase in BMI over the study period^29^.

We extended the results of prior studies in several aspects. First, our study population is three to four times larger than previous studies and considered both men and women. Second, while previous studies considered up to two measures of adiposity per study participant over time, we used for the first time a lifetime approach considering between three to five body silhouettes estimations from childhood to adulthood per participant. Self-reported body silhouettes represent reliable markers of adiposity in the absence of objective measures^30,31^. Accordingly, correlation coefficients from 0.53 to 0.75 have been reported between the recall of body silhouette at different ages (between 5 and 20-year old) and objective measures of BMI at the corresponding ages^30^. Consistently, in the present study, the Pearson correlation coefficient between body silhouettes at age 45 and objectively measured BMI at age 50 was 0.60. On the other hand, body silhouettes also integrate body image perception and may thus bring information beyond adiposity itself. Interestingly, the associations of insomnia symptoms with body silhouette trajectories were independent from BMI. Thus, body silhouette trajectories may potentially add information beyond BMI regarding the likelihood of insomnia symptoms.

The observed associations between body silhouettes trajectories and insomnia outcomes were independent of major confounding variables such as psychological factors. This is of primary importance as strong associations have been highlighted between depression, anxiety and insomnia symptoms^21,22,37^. Taking these psychological factors into account is also important as they may influence body image perception and thus the self-reported body silhouette. Indeed, in our study depression and stress were associated with insomnia symptoms and insomnia syndrome disorder (proxy) together with body silhouettes trajectories. Furthermore, the observed associations with the body silhouette trajectories could reflect associations with BMI at study recruitment on one hand, and/or with the last body silhouette at age 45 on the other hand. However, the results of sensitivity analyses do not support these hypotheses. Finally, consistent results were observed when alternative methods for defining body silhouettes trajectories and for calculating the burden of insomnia symptoms (insomnia symptoms score) were used. Taken together, these aspects support the robustness of the reported associations between body silhouettes trajectories and insomnia outcomes.

Although the pathophysiology of insomnia remains to be established^38^, some mechanisms may contribute to the association between body silhouettes trajectories and insomnia symptoms. For example, increased adiposity over the lifespan could result in over-activity of the hypothalamus-pituitary-adrenal axis and elevated cortisol level, possibly leading to symptoms of insomnia^39,40^. Likewise, the ‘lean-marked increase’ and ‘heavy-stable’ trajectories of body silhouettes may be associated with an underlying chronic inflammation that contributes to insomnia symptoms onset^38^. Furthermore, body silhouettes trajectories may be the results of long-term exposure to behavioral and psychological processes that could explain part of the observed associations, although our analysis was independent of physical activity, depression and stress. In addition, diabetes might be on the path of the association between body silhouette trajectories and insomnia since body silhouette trajectories have been related to incident diabetes and diabetes is known to be associated with insomnia^34,41^. However, the sensitivity analysis did not support this. Last, epigenetics mechanisms might be involved as suggested by preliminary analyses of the methylation status of the circadian genes^42^. However, additional studies are required and the interplay with trajectories of adiposity to be demonstrated.

This study has clinical implications. Self-reported body silhouette is a simple tool that can be easily used in clinical practice, and our findings suggest that it may help to identify subjects at increased odds of developing insomnia symptoms. Weight gain prevention or weight reduction programs could be suggested to subjects presenting with an increasing body silhouette over the lifespan or with a consistently high body silhouette. If replicated, our results are of particular interest for subjects presenting with a marked increased body silhouette during early childhood. It offers the possibility to identify early in life those subjects who are at risk of having insomnia and propose to them adequate interventions to stop weight gain. As recently emphasized in a large population-based study including more than 50,000 children from birth to young adulthood, surveillance for BMI acceleration in childhood is of primary importance, even in the absence of obesity, since this acceleration is directly correlated to later obesity^43^.

We acknowledge the following limitations. First, body silhouettes were assessed retrospectively at study recruitment. This raises the issue of reverse causation, i.e. participants with insomnia symptoms may have been more likely to report at risk body silhouettes at each age. However, the trajectories identified in our study are very similar to the trajectories obtained in studies investigating mortality, cancer or type 2 diabetes^32,33^, making this issue of reverse causation unlikely. Furthermore, the retrospective report of body silhouettes may yield misclassification; in addition, it is well known that obese people tend to underestimate their body silhouette while it is the opposite for underweighted people. As a result, this may attenuate the current association between body silhouettes trajectories and insomnia. Also, information on sleep since childhood would have been useful, but this was not available. Moreover, we cannot rule out that subjects reporting insomnia symptoms tend to systematically report one type of body silhouette more than another. However, the distribution of covariates according to the presence of insomnia symptoms or insomnia disorder (proxy) was in line with previous studies (results not shown), minimizing the possibility of this classification bias. Second, similar to most epidemiological studies^22,29,36^, we accounted for insomnia symptoms and not insomnia disorder as defined by the International Classification of Sleep Disorders^44^. However, we constructed a proxy for insomnia disorder, taking into account EDS. Third, in the absence of polysomnographic measurements, residual confounding by objectively diagnosed other sleep disorders such as SDB cannot be excluded, although we adjusted our analysis on a proxy for SDB. Fourth, the last body silhouette refers to age 45 while age at study recruitment ranged from 50 to 75 years. Thus, we may not have depicted complete body silhouettes trajectories, at least for the oldest participants. Last, the vast majority of the participants are of Caucasian origin; therefore, the generalizability of the results to more ethnically diverse populations needs to be evaluated.

In conclusion, marked increased body silhouette trajectory from childhood to adulthood together with a persistent heavy body silhouette were associated with a higher likelihood of having insomnia symptoms and insomnia disorder (proxy). Self-reported body silhouettes at different ages may represent an easy tool for subjects from the community and physicians to evaluate the risk of insomnia symptoms, and in turn to stimulate behavioral change.

## Material and Methods

### Study population

The design and main objectives of the Paris Prospective Study III (PPS3) have been previously published^45^. The PPS3 is an ongoing prospective observational cohort study on novel markers for the main phenotypes of cardiovascular diseases in mostly healthy subjects. Our study was registered in the World Health Organization international clinical trial registry platform (NCT00741728) and complies with the Declaration of Helsinki. The Ethics Committee of the Cochin Hospital (Paris, France) approved the study protocol and all volunteers signed an informed consent form. Overall, 10 157 men and women aged 50–75 years were recruited at a large preventive medical center, the Centre d’Investigations Préventives et Cliniques (IPC), in Paris (France), between June, 2008 and June, 2012. The IPC is subsidized by the French National Insurance System for Salaried Workers (CNAMTS) and is one of the largest preventive medical centers in France, carrying out 20 to 25 000 free medical examinations per year to all working and retired employees and their families. The standard medical examination included a complete clinical examination including measurement of height, weight and blood pressure, coupled with standard biological tests after an overnight fast. A self-administered questionnaire provided information related to professional activity, lifestyle (tobacco and alcohol consumption, physical activity, diet), personal and family medical history, current health status, and use of medications.

### Body silhouettes

We used body silhouettes adapted from Stunkard^46^. Participants were asked to choose one body silhouette among seven proposed, that best depicted their body silhouette at ages 8, 15, 25, 35 and 45 years. Body silhouettes were presented using children and adult silhouettes, separately for men and women.

### Insomnia symptoms

In a standardized questionnaire, participants self-rated as ‘never’, ‘rarely’, ‘regularly’ or ‘often’ the frequency with which they had 1) difficulties in initiating sleep (DIS), 2) difficulties in maintaining sleep (DMS) and 3) early morning awakening (EMA). These three symptoms are the most common symptoms of insomnia and have been used in several large epidemiologic studies^4,19,21,27,37,47^. Each insomnia symptom was defined as present if rated as ‘regularly’ or ‘often’. We also calculated a score for insomnia symptoms for each participant by summing the weight (i.e. from 0 for ‘never’ to 3 for ‘often’) of self-rated frequency of each insomnia symptom, resulting in a score ranging from 0 to 9 (higher score indicating higher number and frequency of insomnia symptoms).

### Other sleep related variables

#### Excessive daytime sleepiness

Participants were also asked to fill the Epworth Sleepiness Scale (ESS) to assess excessive daytime sleepiness (EDS). The ESS is a self-administered questionnaire containing eight questions evaluating the probability of dozing during daily activities, each question being rated from 0 (would never doze) to 3 (high chance of dozing). The ESS score is the sum of the eight items, ranging from 0 to 24^48^. In accordance with the literature, we considered an ESS score of 11 and above to define EDS^49–54^.

#### Proxy for sleep-disordered breathing

In the absence of an objective measure of sleep disorder breathing (SDB), we used a proxy based on the self-rated frequency of snoring and the level of measured body mass index (BMI)^55^. Snoring rated as regular or often combined with a BMI ≥ 30 kg/m^2^ defined the presence of a possible SDB.

#### Working schedules and sleep related medications

Information on usual working schedules was recorded (i.e. working during the day or night). A medical doctor from the IPC checked current medication during a face-to-face interview with the study participants. To reduce under reporting, participants were asked to come to the IPC with either their most recent medical prescriptions and/or their medication packages. The World Health Organization Anatomical Therapeutic Chemical (ATC) classification was used to categorize medications; in particular sleep-related medications (i.e. those that influence sleep) were identified by ATC code N05 (psycholeptics).

### Outcomes

There were two outcomes. The first considered the presence of at least one insomnia symptom (versus no insomnia symptom). The second considered a proxy of insomnia disorder^44^, which combined the presence of at least one insomnia symptom together with the presence of EDS (to consider possible consequence of insomnia symptoms on daytime activity) (versus the absence of both insomnia symptom and EDS).

### Covariates

Education level was considered as a categorical variable with three categories: 1) did not graduate, 2) less than a high school diploma or, 3) high school diploma and above. Alcohol consumption was categorized as: never, one or two drinks a day and three or more drinks a day. Coffee consumption was categorized as: never, one to four cups a day and five or more cups a day. Depression score was assessed using the 13-items Questionnaire of Depression second version, Abridged (QD2A)^56^. The presence of depressive symptoms was defined by a QD2A score ≥ 7. Stress was estimated using the short version of the Perceived Stress Scale (PSS-4 items)^57^. Prevalence of cardiovascular disease (CVD), including history of stroke, myocardial infarction or angina pectoris, was self-reported. Diabetes was defined by taking blood glucose lowing medication or fasting blood glucose > 7 mmol/L. Hypertension was defined by taking blood pressure lowering medication or systolic blood pressure ≥ 140 mmHg or a diastolic blood pressure ≥ 90 mmHg. We used the validated Baecke score to evaluate physical activity at work, during recreational activities and during sport, a higher score indicating higher level of physical activity^58^. Birth weight was self-reported by the participant on the general health questionnaire in fixed categories including < 2.5 kg, 2.5–4 kg and >4 kg.

### Analytic sample

Among the 10 157 participants included in PPS3, 8 583 participants completed the sleep questionnaires. Subjects with missing data on outcomes (n = 305), exposure (body silhouette trajectory, n = 735) or both (n = 47) were excluded. Hence, 7 496 participants were subsequently included for both main and sensitivity analyses. Comparisons between the included and excluded populations are reported in Table S7.

### Statistical analyses

To construct body silhouette trajectories, we selected subjects reporting their body silhouettes at a minimum at three ages of the five, including the earliest and the latest age, i.e. body silhouettes at age 8 and 45 respectively. We used a group-based trajectory modeling implemented by SAS Proc Traj^59,60^. The optimal number of groups was identified from the change in the Bayesian Information Criterion (BIC) and the average posterior probability (see below). The model including five trajectories presented the best BIC when compared with models including less than 5 trajectories. Furthermore, models including more than 5 trajectories had some average posterior probabilities <20%. Hence, the best model included five clusters of body silhouette trajectories and we named them after their visual aspect (‘lean-stable’, ‘lean-increase’, ‘lean-marked increase’, ‘moderate-stable’ and ‘heavy-stable’, Fig. 1). We then calculated the posterior probability for each participant to belong to one of the five trajectories and attributed each participant to a trajectory according to the highest probability among the five calculated. The average posterior probabilities for each trajectory were 86%, 88%, 79%, 89% and 91%, indicating very good allocation of subjects to the trajectories.

To test the reliability of the obtained trajectories, we also modeled trajectories using a different approach. We used a package implemented in R software (kml package) based on an implementation of k-means clustering algorithm adapted for longitudinal data^61^. According to quality criteria (Calinski & Harabasz, Ray & Turi and Davies & Bouldin), the optimal number of trajectories was five. Using this approach, we found very similar body silhouette trajectories as the ones previously described above (Fig. S1).

Logistic regression was used to quantify the relationship between body silhouette trajectory (using the ‘lean-stable’ trajectory as the reference exposure category) and a) the presence of at least one insomnia symptom (versus 0), and b) the proxy for insomnia disorder (versus no proxy for insomnia disorder). Results were expressed as odds ratio (OR) and 95% confidence intervals (CI). Unadjusted and adjusted ORs are provided. Regression models were adjusted for potential confounding factors such as age, sex, coffee and alcohol consumption, education level, physical activity, living alone status, depression, stress and sleep related medications. Considering that there are gender differences in the prevalence of insomnia, we then conducted the previous analyses separately in men and women.

Several supplementary analyses were conducted. First, we investigated the associations between the last body silhouette (at age 45) and insomnia symptoms and insomnia disorder (proxy) and made qualitative comparisons with associations observed with body silhouette trajectories. Second, we adjusted the regression models for a proxy for SDB as defined previously. Third, we quantified the association between body silhouette trajectories and the score of insomnia symptoms as defined previously using linear regression analysis. Fourth, to assess to which extent body silhouette trajectories provide information beyond BMI, we further adjusted our analysis for BMI at study recruitment. Fifth, because birth weight may influence body shape trajectories, we further adjusted our analysis on birth weight. Sixth, because type 2 diabetes might be on the path of the association between body silhouette trajectories and insomnia^34,41^, we further adjusted the analysis on type 2 diabetes.

All analyses were two-sided and we considered a p-value < 0.05 as statistically significant. Statistical analyses were performed with R version 3.1.3 (www.r-project.org) and SAS version 9.3 (SAS Institute Inc., Cary, NC, USA).

## Supplementary information

supplementary material

## Data Availability

Data may be available on request to the last author.

## References

[1] Soldatos CR, Allaert FA, Ohta T, Dikeos DG (2005). How do individuals sleep around the world? Results from a single-day survey in ten countries. Sleep Med..

[2] Léger D, Bayon V (2010). Societal costs of insomnia. Sleep Med. Rev..

[3] Schutte-Rodin S, Broch L, Buysse D, Dorsey C, Sateia M (2008). Clinical guideline for the evaluation and management of chronic insomnia in adults. J. Clin. Sleep Med..

[4] Ohayon MM (2002). Epidemiology of insomnia: what we know and what we still need to learn. Sleep Med. Rev..

[5] Morin CM, LeBlanc M, Daley M, Gregoire JP, Mérette C (2006). Epidemiology of insomnia: prevalence, self-help treatments, consultations, and determinants of help-seeking behaviors. Sleep Med..

[6] Ford, E. S., Cunningham, T. J., Giles, W. H. & Croft, J. B. Trends in insomnia and excessive daytime sleepiness among U.S. adults from 2002 to 2012. *Sleep Med*. **16**, 372–378 (2015). 10.1016/j.sleep.2014.12.008 PMC4763609 25747141

[7] Kronholm E (2008). Trends in self-reported sleep duration and insomnia-related symptoms in Finland from 1972 to 2005: a comparative review and re-analysis of Finnish population samples. J. Sleep Res..

[8] Pallesen S, Sivertsen B, Nordhus IH, Bjorvatn B (2014). A 10-year trend of insomnia prevalence in the adult Norwegian population. Sleep Med..

[9] Javaheri S, Redline S (2017). Insomnia and risk of cardiovascular disease. Chest.

[10] Li Y (2014). Association between insomnia symptoms and mortality: a prospective study of U.S. men. Circulation.

[11] He Q, Zhang P, Li G, Dai H, Shi J (2017). The association between insomnia symptoms and risk of cardio-cerebral vascular events: A meta-analysis of prospective cohort studies. Eur. J. Prev. Cardiol..

[12] Edinger JD (2004). Derivation of research diagnostic criteria for insomnia: report of an American Academy of Sleep Medicine Work Group. Sleep.

[13] Léger D (2014). Insomnia and accidents: cross-sectional study (EQUINOX) on sleep-related home, work and car accidents in 5293 subjects with insomnia from 10 countries. J. Sleep Res..

[14] Philip P (2006). Insomniac complaints interfere with quality of life but not with absenteeism: respective role of depressive and organic comorbidity. Sleep Med..

[15] Sivertsen B (2006). The long-term effect of insomnia on work disability: the HUNT-2 historical cohort study. Am. J. Epidemiol..

[16] Kay-Stacey M, Attarian H (2016). Advances in the management of chronic insomnia. BMJ.

[17] Sateia MJ, Buysse DJ, Krystal AD, Neubauer DN, Heald JL (2017). Clinical Practice Guideline for the Pharmacologic Treatment of Chronic Insomnia in Adults: An American Academy of Sleep Medicine Clinical Practice Guideline. J. Clin. Sleep Med..

[18] Klink ME, Quan SF, Kaltenborn WT, Lebowitz MD (1992). Risk factors associated with complaints of insomnia in a general adult population. Influence of previous complaints of insomnia. Arch. Intern. Med..

[19] Ancoli-Israel S, Roth T (1999). Characteristics of insomnia in the United States: results of the 1991 National Sleep Foundation Survey. I. Sleep.

[20] Leger D, Guilleminault C, Dreyfus JP, Delahaye C, Paillard M (2000). Prevalence of insomnia in a survey of 12,778 adults in France. J. Sleep Res..

[21] Sivertsen B, Krokstad S, Øverland S, Mykletun A (2009). The epidemiology of insomnia: associations with physical and mental health. The HUNT-2 study. J. Psychosom. Res..

[22] Taylor DJ, Lichstein KL, Durrence HH, Reidel BW, Bush AJ (2005). Epidemiology of insomnia, depression, and anxiety. Sleep.

[23] Bixler EO (2005). Excessive daytime sleepiness in a general population sample: the role of sleep apnea, age, obesity, diabetes, and depression. J. Clin. Endocrinol. Metab..

[24] Vgontzas AN (1998). Obesity without sleep apnea is associated with daytime sleepiness. Arch. Intern. Med..

[25] Jordan AS, McSharry DG, Malhotra A (2014). Adult obstructive sleep apnoea. The Lancet.

[26] Lallukka T, Haario P, Lahelma E, Rahkonen O (2012). Associations of relative weight with subsequent changes over time in insomnia symptoms: a follow-up study among middle-aged women and men. Sleep Med..

[27] Jaussent I (2011). Insomnia symptoms in older adults: associated factors and gender differences. Am. J. Geriatr. Psychiatry.

[28] Janson C, Lindberg E, Gislason T, Elmasry A, Boman G (2001). Insomnia in men-a 10-year prospective population based study. Sleep.

[29] LeBlanc M (2009). Incidence and risk factors of insomnia in a population-based sample. Sleep.

[30] Must A, Willett WC, Dietz WH (1993). Remote recall of childhood height, weight, and body build by elderly subjects. Am. J. Epidemiol..

[31] Tehard B, van Liere MJ, Com Nougué C, Clavel-Chapelon F (2002). Anthropometric measurements and body silhouette of women: validity and perception. J. Am. Diet. Assoc..

[32] Song M (2016). Trajectory of body shape in early and middle life and all cause and cause specific mortality: results from two prospective US cohort studies. BMJ.

[33] Song M (2016). Trajectory of body shape across the lifespan and cancer risk. Int. J. Cancer.

[34] Fagherazzi G (2015). The association of body shape trajectories over the life course with type 2 diabetes risk in adulthood: a group-based modeling approach. Ann. Epidemiol..

[35] Fagherazzi G, Guillas G, Boutron-Ruault M-C, Clavel-Chapelon F, Mesrine S (2013). Body shape throughout life and the risk for breast cancer at adulthood in the French E3N cohort. Eur. J. Cancer Prev..

[36] Singareddy R (2012). Risk factors for incident chronic insomnia: a general population prospective study. Sleep Med..

[37] Neckelmann D, Mykletun A, Dahl AA (2007). Chronic insomnia as a risk factor for developing anxiety and depression. Sleep.

[38] Levenson JC, Kay DB, Buysse DJ (2015). The pathophysiology of insomnia. Chest.

[39] Xia L, Chen G-H, Li Z-H, Jiang S, Shen J (2013). Alterations in hypothalamus-pituitary-adrenal/thyroid axes and gonadotropin-releasing hormone in the patients with primary insomnia: a clinical research. PloS One.

[40] Incollingo Rodriguez AC (2015). Hypothalamic-pituitary-adrenal axis dysregulation and cortisol activity in obesity: A systematic review. Psychoneuroendocrinology.

[41] Vgontzas AN (2009). Insomnia with objective short sleep duration is associated with type 2 diabetes: A population-based study. Diabetes Care.

[42] Barclay NL, Gregory AM (2013). Quantitative genetic research on sleep: a review of normal sleep, sleep disturbances and associated emotional, behavioural, and health-related difficulties. Sleep Med. Rev..

[43] Geserick M (2018). Acceleration of BMI in Early Childhood and Risk of Sustained Obesity. N. Engl. J. Med..

[44] American Academy of Sleep Medicine. International Classification of Sleep Disorders. 3rd ed. Darien, IL: American Academy of Sleep Medicine. (2014).

[45] Empana J-P (2011). Paris Prospective Study III: a study of novel heart rate parameters, baroreflex sensitivity and risk of sudden death. Eur. J. Epidemiol..

[46] Stunkard AJ, Sørensen T, Schulsinger F (1983). Use of the Danish Adoption Register for the study of obesity and thinness. Res. Publ. - Assoc. Res. Nerv. Ment. Dis..

[47] Olson LG (1996). A community survey of insomnia in Newcastle. Aust. N. Z. J. Public Health.

[48] Kendzerska TB, Smith PM, Brignardello-Petersen R, Leung RS, Tomlinson GA (2014). Evaluation of the measurement properties of the Epworth sleepiness scale: a systematic review. Sleep Med. Rev..

[49] Johns M, Hocking B (1997). Daytime sleepiness and sleep habits of Australian workers. Sleep.

[50] Walsleben JA (2004). Sleep and reported daytime sleepiness in normal subjects: the Sleep Heart Health Study. Sleep.

[51] Boden-Albala B (2012). Daytime sleepiness and risk of stroke and vascular disease: findings from the Northern Manhattan Study (NOMAS). Circ. Cardiovasc. Qual. Outcomes.

[52] Hayley AC (2015). Excessive daytime sleepiness and metabolic syndrome: a cross-sectional study. Metabolism..

[53] Goldstein IB, Ancoli-Israel S, Shapiro D (2004). Relationship between daytime sleepiness and blood pressure in healthy older adults. Am. J. Hypertens..

[54] Hayley AC (2014). Prevalence of excessive daytime sleepiness in a sample of the Australian adult population. Sleep Med..

[55] Young T (1993). The occurrence of sleep-disordered breathing among middle-aged adults. N. Engl. J. Med..

[56] Pichot, P. A self-report inventory on depressive symptomatology (QD2) and its abridged form (QD2). In Assessment of Depression. Springer 108–22 (1986).

[57] Ingram PB, Clarke E, Lichtenberg JW (2016). Confirmatory Factor Analysis of the Perceived Stress Scale-4 in a Community Sample. Stress Health J. Int. Soc. Investig. Stress.

[58] Baecke JA, Burema J, Frijters JE (1982). A short questionnaire for the measurement of habitual physical activity in epidemiological studies. Am. J. Clin. Nutr..

[59] Jones B, Nagin D, Roeder KA (2001). SAS procedure based on mixture models for estimating developmental trajectories. Sociol Methods Res.

[60] Jones BL, Nagin DS (2007). Advances in Group-Based Trajectory Modeling and an SAS Procedure for Estimating Them. Sociol. Methods Res..

[61] Genolini C, Falissard B (2011). KmL: a package to cluster longitudinal data. Comput. Methods Programs Biomed..

