# Supplementary material for: Body Silhouette Trajectories Over the Lifespan and Insomnia Symptoms: The Paris Prospective Study 3
Source: Sci Rep. 2019 Feb 7;9:1581. doi: 10.1038/s41598-018-38145-7 (PMC6367427; doi:10.1038/s41598-018-38145-7)
Supplement: Supplementary file 1 — supplementary material [file 41598_2018_38145_MOESM1_ESM.docx]

**Supplementary material**

**Title:** Body Silhouette Trajectories Over the Lifespan and Insomnia Symptoms: The Paris Prospective Study 3.

**Authors:** Q. Lisan, M. Tafflet, F. Thomas, P. Boutouyrie, C. Guibout, J. Haba-Rubio, R. Climie, MC. Périer, T. Van Sloten, B. Pannier, P. Marques-Vidal, X. Jouven, JP. Empana

Supplementary figure 1: body silhouette trajectories obtained using a k-means approach.

Supplementary table 1: association of body silhouette trajectories and study covariates with insomnia symptoms and insomnia disorder (proxy).

|  | Outcome | | | | |
| --- | --- | --- | --- | --- | --- |
|  | ≥1 symptom versus 0 | |  | Proxy for insomnia disorder (≥1 symptom and EDS) | |
|  | Unadjusted  OR (95% CI) | Fully adjusted  OR (95% CI) |  | Unadjusted  OR (95% CI) | Fully adjusted  OR (95% CI) |
| ***Body silhouette trajectory*** |  |  |  |  |  |
| Lean-stable | 1 (reference) | 1 (reference) |  | 1 (reference) | 1 (reference) |
| Lean-increase | 1.13 (0.96 – 1.33) | 1.17 (0.97 – 1.42) |  | 1.15 (0.89 – 1.47) | 1.10 (0.81 – 1.48) |
| Lean-marked increase | 1.21 (1.05 – 1.40) | 1.36 (1.15 – 1.61) |  | 1.60 (1.29 – 1.96) | 1.67 (1.32 – 2.12) |
| Moderate-stable | 1.08 (0.96 – 1.21) | 1.09 (0.95 – 1.25) |  | 1.14 (0.95 – 1.36) | 1.08 (0.88 – 1.34) |
| Heavy-stable | 1.36 (1.13 – 1.64) | 1.28 (1.03 – 1.58) |  | 1.75 (1.35 – 2.24) | 1.50 (1.12 – 2.01) |
| Male gender |  | 0.66 (0.58 – 0.75) |  |  | 1.02 (0.84 – 1.22) |
| Age (per 1 year increase) |  | 1.04 (1.03 – 1.05) |  |  | 0.98 (0.96 – 0.99) |
| Education level |  |  |  |  |  |
| No graduation |  | 1 (reference) |  |  | 1 (reference) |
| Under HSD |  | 0.92 (0.67 – 1.26) |  |  | 0.90 (0.58 – 1.43) |
| ≥ HSD |  | 1.01 (0.74 – 1.37) |  |  | 1.01 (0.67 – 1.57) |
| Coffee consumption |  |  |  |  |  |
| Never |  | 1 (reference) |  |  | 1 (reference) |
| 1 – 4 cups a day |  | 0.90 (0.75 – 1.04) |  |  | 1.04 (0.83 – 1.31) |
| ≥ 5 cups a day |  | 1.04 (0.81 – 1.32) |  |  | 1.32 (0.94 – 1.85) |
| Alcohol consumption |  |  |  |  |  |
| Never |  | 1 (reference) |  |  | 1 (reference) |
| 1 – 2 drinks a day |  | 1.06 (0.88 – 1.28) |  |  | 0.90 (0.69 – 1.18) |
| ≥ 3 drinks a day |  | 1.29 (1.02 – 1.63) |  |  | 1.03 (0.74 – 1.45) |
| Living alone |  | 0.86 (0.76 – 0.99) |  |  | 0.94 (0.77 – 1.14) |
| Physical activity score |  | 0.95 (0.91 – 0.98) |  |  | 0.99 (0.93 – 1.04) |
| Depression |  | 1.85 (1.45 – 2.37) |  |  | 1.68 (1.28 – 2.19) |
| Stress * |  | 1.05 (1.03 – 1.07) |  |  | 1.07 (1.04 – 1.11) |
| Use of sleep related medications |  | 2.96 (2.10 – 4.29) |  |  | 1.11 (0.76 – 1.57) |

*Abbreviations:* OR: odd ratio, CI: confidence interval, vs: versus, ref: reference category, HSD: high school diploma, EDS: excessive daytime sleepiness.

*Note:* models are adjusted for the listed covariates. *: per 1 point increase in the score of stress (PSS-4).

Supplementary table 2: sex-stratified analyses of the association of body silhouette trajectories with insomnia symptoms and insomnia disorder (proxy).

|  | Outcome | | | | |
| --- | --- | --- | --- | --- | --- |
|  | ≥1 symptom versus 0 | |  | Proxy for insomnia disorder (≥1 symptom and EDS) | |
|  | Unadjusted | Fully adjusted |  | Unadjusted | Fully adjusted |
| ***Men: body silhouette trajectory*** |  |  |  |  |  |
| Lean-stable | 1 (reference) | 1 (reference) |  | 1 (reference) | 1 (reference) |
| Lean-increase | 1.14 (0.94 – 1.39) | 1.15 (0.92 – 1.45) |  | 1.19 (0.87 – 1.62) | 1.12 (0.77 – 1.59) |
| Lean-marked increase | 1.22 (1.03 – 1.45) | 1.25 (1.03 – 1.52) |  | 1.66 (1.29 – 2.15) | 1.64 (1.23 – 2.20) |
| Moderate-stable | 1.07 (0.92 – 1.24) | 1.07 (0.90 – 1.27) |  | 1.24 (0.98 – 1.58) | 1.14 (0.86 – 1.51) |
| Heavy-stable | 1.22 (0.94 – 1.58) | 1.15 (0.86 – 1.54) |  | 1.78 (1.23 – 2.55) | 1.78 (1.18 – 2.64) |
|  |  |  |  |  |  |
| ***Women: body silhouette trajectory*** |  |  |  |  |  |
| Lean-stable | 1 (reference) | 1 (reference) |  | 1 (reference) | 1 (reference) |
| Lean-increase | 1.32 (0.96 – 1.82) | 1.20 (0.83 – 1.73) |  | 1.11 (0.70 – 1.72) | 1.11 (0.63 – 1.89) |
| Lean-marked increase | 1.57 (1.17 – 2.13) | 1.75 (1.24 – 2.49) |  | 1.55 (1.06 – 2.25) | 1.83 (1.19 – 2.79) |
| Moderate-stable | 1.00 (0.83 – 1.20) | 1.14 (0.91 – 1.41) |  | 1.00 (0.75 – 1.32) | 1.00 (0.71 – 1.40) |
| Heavy-stable | 1.31 (1.00 – 1.74) | 1.42 (1.03 – 1.94) |  | 1.64 (1.14 – 2.33) | 1.24 (0.80 – 1.89) |

*Note:* fully adjusted models are adjusted for age, education level, coffee and alcohol consumptions, living alone, physical activity, depression, stress and sleep related medications.

Supplementary table 3: associations between body silhouette trajectories and each insomnia symptom.

|  | Outcome: | | |
| --- | --- | --- | --- |
|  | Difficulty in initiating sleep  OR (95% CI) | Difficulty in maintaining sleep  OR (95% CI) | Early morning awakening  OR (95% CI) |
| ***Body silhouette trajectory*** |  |  |  |
| Lean-stable | 1 (reference) | 1 (reference) | 1 (reference) |
| Lean-increase | 1.30 (1.01 – 1.66) | 1.23 (0.99 – 1.51) | 1.19 (0.98 – 1.44) |
| Lean-marked increase | 1.20 (0.96 – 1.49) | 1.45 (1.21 – 1.73) | 1.24 (1.06 – 1.46) |
| Moderate-stable | 0.97 (0.81 – 1.17) | 1.06 (0.91 – 1.23) | 1.16 (1.02 – 1.32) |
| Heavy-stable | 0.99 (0.76 – 1.29) | 1.16 (0.92 – 1.44) | 1.21 (0.98 – 1.48) |

*Note:* models are adjusted for age, sex, education level, coffee and alcohol consumptions, living alone, physical activity, depression, stress and the use of sleep related medications.

*Abbreviations*: OR: odd ratio, CI: confidence interval.

Supplementary table 4: separate association between the last body silhouette and insomnia symptoms or insomnia disorder (proxy).

|  | Outcome | |  |
| --- | --- | --- | --- |
|  | ≥1 symptom  OR (95% CI) | Proxy for insomnia disorder (≥1 symptom and EDS)  OR (95% CI) |  |
| ***Exposure:*** Body silhouette at age 45 |  |  |  |
| A (n=43) | 1.05 (0.46 – 2.61) | 0.58 (0.09 – 2.02) |  |
| B (n=478) | 0.96 (0.76 – 1.21) | 0.81 (0.54 – 1.18) |  |
| C (n=1 794) | 0.88 (0.76 – 1.02) | 0.83 (0.66 – 1.05) |  |
| D (n=2 825) | 1 (reference) | 1 (reference) |  |
| E (n=1 821) | 1.16 (1.01 – 1.34) | 1.23 (1.01 – 1.51) |  |
| F (n=477) | 1.14 (0.90 – 1.43) | 1.27 (0.92 – 1.73) |  |
| G (n=58) | 2.20 (1.14 – 4.59) | 2.67 (1.33 – 5.07) |  |

*Note:* models are adjusted for age, sex, education level, coffee and alcohol consumptions, living alone, physical activity, depression, stress and the use of sleep related medications.

*Abbreviations*: EDS: excessive daytime sleepiness, OR: odd ratio, CI: confidence interval.

Supplementary table 5: association of body silhouette trajectories with the score of insomnia symptoms.

|  | Coefficient | p-value |  |
| --- | --- | --- | --- |
| ***Body silhouette trajectory*** |  |  |  |
| Lean-stable | Ref | Ref |  |
| Lean-increase | 0.24 | 0.004 |  |
| Lean-marked increase | 0.25 | 0.0006 |  |
| Moderate-stable | 0.07 | 0.23 |  |
| Heavy-stable | 0.24 | 0.006 |  |

*Abbreviations:* ref: reference category.

*Note:* regression coefficients were obtained using linear multivariable regression analysis, adjusted for age, sex, education level, coffee and alcohol consumptions, living alone, physical activity, depression, stress and the use of sleep related medications.

Supplementary table 6: multivariate associations of body silhouette trajectories for insomnia symptoms and insomnia disorder (proxy): without and with adjustment for categories of body mass index at study recruitment.

|  | Outcome | | | | |
| --- | --- | --- | --- | --- | --- |
|  | ≥1 symptom versus 0 | |  | Proxy for insomnia disorder (≥1 symptom and EDS) | |
|  | Fully adjusted | Fully adjusted + BMI |  | Fully adjusted | Fully adjusted + BMI |
| ***Body silhouette trajectory*** |  |  |  |  |  |
| Lean-stable | 1 (reference) | 1 (reference) |  | 1 (reference) | 1 (reference) |
| Lean-increase | 1.17 (0.97 – 1.42) | 1.17 (0.96– 1.42) |  | 1.10 (0.81 – 1.48) | 1.09 (0.81 – 1.47) |
| Lean-marked increase | 1.36 (1.15 – 1.61) | 1.35 (1.14 – 1.61) |  | 1.67 (1.32 – 2.12) | 1.55 (1.21 – 1.98) |
| Moderate-stable | 1.09 (0.95 – 1.25) | 1.09 (0.95 – 1.25) |  | 1.08 (0.88 – 1.34) | 1.04 (0.83 – 1.29) |
| Heavy-stable  Body mass index | 1.28 (1.03 – 1.58) | 1.26 (1.01 – 1.57) |  | 1.50 (1.12 – 2.01) | 1.32 (0.96 – 1.79) |
| <18.5 kg/m^2^ | - | 0.97 (0.61 – 1.58) |  | - | 1.10 (0.52 – 2.08) |
| 18.5 – 25 kg/m^2^ | - | 1 (reference) |  | - | 1 (reference) |
| 25 – 30 kg/m^2^ | - | 0.97 (0.86 – 1.09) |  | - | 1.12 (0.93 – 1.35) |
| >30 kg/m^2^ | - | 1.07 (0.87 – 1.32) |  | - | 1.49 (1.12 – 1.95) |

*Abbreviations:* OR: odd ratio, CI: confidence interval, vs: versus, BMI: body mass index.

*Note:* fully adjusted models are adjusted for age, sex, education level, coffee and alcohol consumptions, living alone, physical activity, depression, stress and sleep medications.

Supplementary table 7: comparison between included and excluded study participants.

|  | Included  N=7,496 | Excluded  N=1,087 | p-value |
| --- | --- | --- | --- |
| ≥1insomnia symptom | 4 616 (61.5) | 555 (56.2) | 0.002 |
| Proxy for insomnia disorder  (≥1 insomnia symptom and EDS) | 877 (11.7) | 98 (9.0) | 0.01 |
| Body silhouette trajectory |  |  | 0.089 |
| Lean-stable | 2 438 (32.5) | 74 (26.9) |  |
| Lean-increase | 823 (11.0) | 40 (14.5) |  |
| Lean-marked increase | 1 176 (15.7) | 53 (19.3) |  |
| Moderate-stable | 2 449 (32.7) | 88 (32.0) |  |
| Heavy-stable | 610 (8.14) | 20 (7.27) |  |
| Male gender | 4 610 (61.5) | 602 (55.4) | <0.001 |
| Age (years) | 59.6 ± 6.21 | 61.4 ± 6.58 | <0.001 |
| Education level |  |  | <0.001 |
| No graduation | 255 (3.43) | 141 (13.2) |  |
| Under high school diploma | 1 692 (22.8) | 372 (34.7) |  |
| ≥ high school diploma | 5 482 (73.8) | 559 (52.1) |  |
| Current smoker | 1 023 (13.7) | 164 (15.1) | 0.226 |
| Coffee consumption |  |  | 0.060 |
| Never | 1 196 (16.1) | 204 (18.9) |  |
| 1 – 4 cups a day | 5 661 (76.0) | 793 (73.6) |  |
| ≥ 5 cups a day | 589 (7.91) | 81 (7.51) |  |
| Depression | 618 (8.28) | 91 (8.40) | 0.938 |
| Stress (PSS4 score) | 3.75 ± 2.73 | 4.26 ± 2.78 | <0.001 |
| Living alone | 1 817 (24.3) | 333 (30.7) | <0.001 |
| Alcohol consumption |  |  | <0.001 |
| 1 – 2 drinks per day | 5 669 (76.8) | 758 (70.8) |  |
| ≥ 3 drinks per day | 952 (12.9) | 131 (12.2) |  |
| Never | 762 (10.3) | 181 (16.9) |  |
| Score of physical activity | 6.23 ± 1.43 | 6.12 ± 1.40 | 0.019 |
| Prevalent CVD | 144 (1.92) | 44 (2.22) | 0.596 |
| Hypertension | 2 525 (33.9) | 424 (39.3) | 0.001 |
| Diabetes | 254 (3.40) | 62 (5.72) | <0.001 |
| EDS | 1 126 (16.4) | 132 (13.4) | 0.02 |
| Night working | 113 (1.51) | 24 (2.21) | 0.113 |
| SDB (proxy) | 329 (4.41) | 42 (3.95) | 0.547 |
| Use of sleep medication | 271 (4.59) | 45 (5.43) | 0.326 |
| Body mass index | 25.1 ± 3.61 | 25.4 ± 3.86 | 0.014 |

*Abbreviations:* HSD: high school diploma, CVD: cardiovascular disease, EDS: excessive daytime sleepiness, SDB: sleep disordered breathing.

*Note:* values are number of participants (percentages) or mean ± standard deviation.
